# *Lotus japonicus* alters *in planta* fitness of *Mesorhizobium loti* dependent on symbiotic nitrogen fixation

**DOI:** 10.1371/journal.pone.0185568

**Published:** 2017-09-28

**Authors:** Kenjiro W. Quides, Glenna M. Stomackin, Hsu-Han Lee, Jeff H. Chang, Joel L. Sachs

**Affiliations:** 1 Department of Evolution, Ecology, and Organismal Biology, University of California, Riverside, CA, United States of America; 2 Department of Botany and Plant Pathology, Oregon State University, Corvallis, OR, United States of America; 3 Department of Botany and Plant Sciences, University of California, Riverside, CA, United States of America; 4 Institute for Integrative Genome Biology, University of California, Riverside, CA, United States of America; Oklahoma State University, UNITED STATES

## Abstract

Rhizobial bacteria are known for their capacity to fix nitrogen for legume hosts. However ineffective rhizobial genotypes exist and can trigger the formation of nodules but fix little if any nitrogen for hosts. Legumes must employ mechanisms to minimize exploitation by the ineffective rhizobial genotypes to limit fitness costs and stabilize the symbiosis. Here we address two key questions about these host mechanisms. What stages of the interaction are controlled by the host, and can hosts detect subtle differences in nitrogen fixation? We provide the first explicit evidence for adaptive host control in the interaction between *Lotus japonicus* and *Mesorhizobium loti*. In both single inoculation and co-inoculation experiments, less effective rhizobial strains exhibited reduced *in planta* fitness relative to the wildtype *M*. *loti*. We uncovered evidence of host control during nodule formation and during post-infection proliferation of symbionts within nodules. We found a linear relationship between rhizobial fitness and symbiotic effectiveness. Our results suggest that *L*. *japonicus* can adaptively modulate the fitness of symbionts as a continuous response to symbiotic nitrogen fixation.

## Introduction

Rhizobial bacteria instigate the formation of root nodules on legumes where the bacteria fix atmospheric nitrogen for the host, a process that can greatly enhance plant growth and fitness [1]. In exchange for fixed nitrogen the plant provides photosynthates that allow rhizobia to proliferate *in planta* and after nodule senescence, replenish the soil [2]. However, legumes often encounter ineffective rhizobia that trigger the formation of nodules but fix little if any nitrogen [3–16]. By not providing fixed nitrogen ineffective rhizobia have the potential to exploit resources from the host without paying the high energetic expense of fixing nitrogen [15,17–20]. To maximize net fitness benefits of symbiosis, legumes must exhibit ‘host control’ traits that constrain the effects of ineffective rhizobial genotypes and select against symbiont exploitation [17,21,22].

There are two stages of the interaction where legumes have the potential to bias investment towards beneficial rhizobia and limit physiological inputs into ineffective genotypes. In a ‘partner choice’ model, legumes could discriminate between different genotypes of rhizobia during the process of nodule organogenesis [23–25]. Evidence suggests legumes restrict certain genotypes of rhizobia at the early stages of nodule development [26–30] or block nodulation caused by toxin-producing rhizobia [31,32]. However, it is unclear whether nitrogen fixation is expressed early in the process for legumes to discriminate between effective and ineffective genotypes [33–42]. In a ‘sanctions’ model, after nodule organogenesis is complete, legumes have the potential to selectively target rhizobia that fail to fix sufficient amounts of nitrogen within the nodule and reduce their fitness relative to beneficial genotypes [17,21,22]. Several experiments have demonstrated that nodules with nitrogen-fixing rhizobia grow (and the rhizobia within rapidly proliferate) whereas nodules with ineffective rhizobia tend to stay small (and the rhizobia within have reduced fitness) [43–47]. Some experiments have failed to find evidence for sanctions but this could be a consequence of challenges in controlling for variation in competitive ability and host specificity among rhizobial genotypes [39,48–50]. Experiments have found evidence for partner choice and/or sanctions in diverse legumes [34,35,40,43,45–49,51–54]. While the molecular dialogue that occurs prior to nodule formation has been well characterized [23,55], the triggers for sanctions have received little attention [45,51].

*Mesorhizobium loti* MAFF303099 (MAFF) fixes nitrogen for the host *Lotus japonicus*, a perennial herbaceous diploid, allowing hosts to grow without any other source of nitrogen [56]. *L*. *japonicus* forms determinate nodules that lack a continuous meristem. *L*. *japonicus* nodules have a relatively homogenous population of differentiated intracellular rhizobia (i.e., bacteroids), cease growth after nodule development is complete, and because the bacteroids do not terminally differentiate, allow rhizobia to escape back into the soil during nodule senescence. This is in contrast to other legumes such as *Medicago truncatula* that form indeterminate nodules that grow throughout the functional association, and have a spatial gradation of bacteroids in different developmental stages. The bacteroids of indeterminate nodules tend to terminally differentiate and cannot escape the nodule, but nonetheless a subset of viable rhizobia can be released upon nodule senescence [2,57,58].

In the *Lotus*-*Mesorhizobium* symbiosis both symbiotic partners have been developed as models for the molecular and cellular basis of nodulation [59,60]. Mutants of MAFF have been generated by signature-tagged mutagenesis and their insertion sites have been identified [61]. Several mutants affected in nitrogen fixation have been identified. STM30 has a transposon inserted in mll0343, encoding glutamine synthetase I (strain ID 10T05g06). The mutant is significantly reduced in its ability to fix nitrogen, as measured by acetylene reduction (ca. 45%, [62]). Glutamine synthetase (along with glutamine synthase) is responsible for the assimilation of ammonium in legume nodules, which is the main product of nitrogen fixation [62]. The MAFF mutant STM6 has a transposon inserted in mlr5906, which is the nitrogenase gene *nifD* (strain ID 17T02d02). This mutant is incapable of fixing nitrogen [61]. Nitrogenases are a family of metalloenzymes that catalyze the reduction of dinitrogen to ammonia. In MAFF, the majority of the genes necessary for nodulation and nitrogen fixation are clustered in a symbiosis island [59].

The goals of our experiment were to (i) test whether mildly effective and ineffective mutants of *M*. *loti* nodulate *L*. *japonicus* hosts at reduced rates when competing against wildtype MAFF, (ii) examine whether the MAFF mutants are reduced in population sizes within singly infected and co-infected host nodules, and (iii) investigate the triggering of sanctions in response to varying amounts of nitrogen fixation. We inoculated *L*. *japonicus* hosts with single and mixed inocula of MAFF, STM30, and STM6 in order to estimate symbiotic effectiveness (effects on host growth in the absence of other nitrogen sources), host investment in symbionts (nodule biomass), and symbiont fitness (viable within-nodule population size). Additionally, we tested models of nodulation rates for each strain and host response to rhizobia that differ in symbiotic effectiveness.

## Results

### Host and symbiont growth in a controlled environment

**Fig 1:**
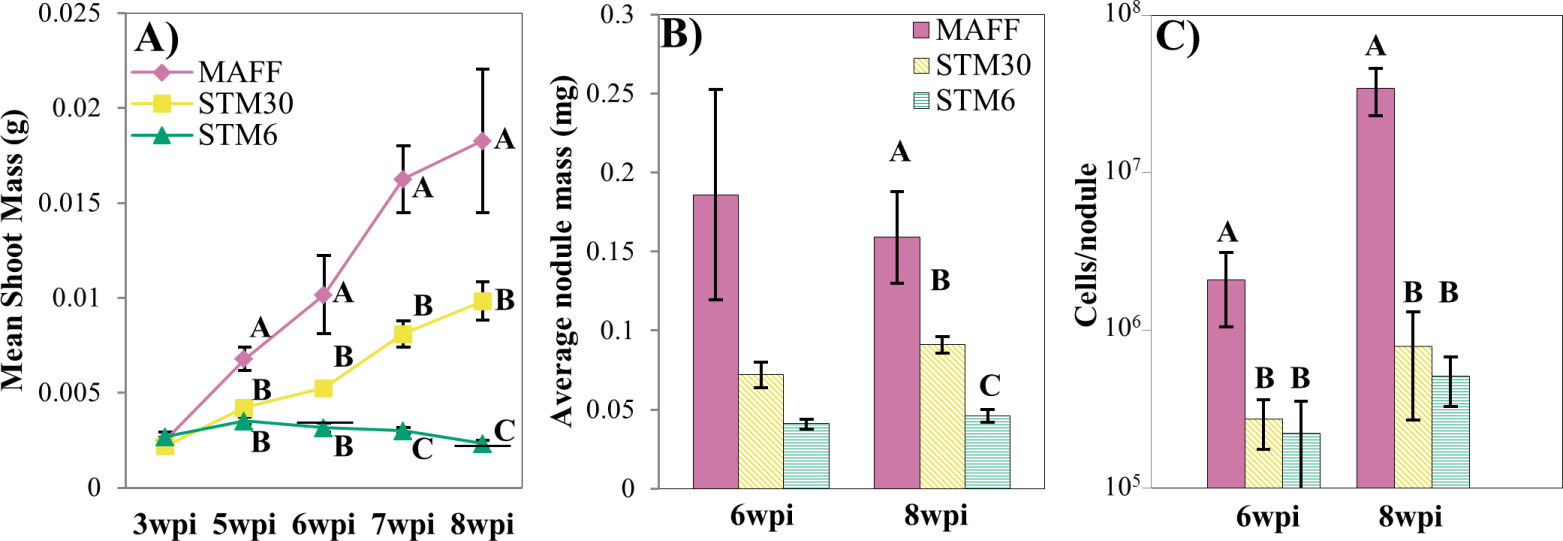
Traits of host and symbiont in growth chamber experiment. *L*. *japonicus* hosts were inoculated with near-isogenic *M*. *loti* strains MAFF, STM30, and STM6 and host and symbiont phenotypes were measured at times indicated. (**A)** Symbiont effectiveness was measured as dried shoot biomass. (**B)** Host investment in symbionts was measured as average individual biomass of nodules (dry weight). (**C)** Rhizobial fitness was estimated based on CFUs from serially diluted extracts of crushed nodules. Error bars indicate one standard error from the mean. Horizontal lines **(A)** represent the mean dried shoot biomass for uninoculated controls at 6 (0.003g) and 8wpi (0.002g). Data points demarked with different letters indicate significant differences between strains at a given wpi. (one-way ANOVA, post-hoc Student’s t-test, α = 0.05).

MAFF, STM30, and STM6 were singly inoculated on *L*. *japonicus* seedlings growing in sterilized growth pouches filled with N-free Jensen’s fertilizer and in an environmentally-controlled setting [63]. Experimental treatments each began with 20 seeds, but after germination the number of surviving plants ranged from 10 to 18 (S1 Table). Plants were harvested from 3 weeks post infection (wpi) to 8wpi. These time points were selected based on data from pilot experiments and visualization of key morphological differences, such as observable differences in nodule size, flowering, and senescence. At the earliest time point, there were no detectable differences in shoot biomass between inoculation treatments (ANOVA, F_2,38_ = 0.994, *P* = 0.380; Fig 1A). By 5wpi, a significant difference was observed, with MAFF-inoculated plants having significantly greater shoot biomass than hosts inoculated with STM30 or STM6, and this pattern was maintained at 6wpi (5wpi: ANOVA, F_2,44_ = 19.052, *P* < 0.001; 6wpi: ANOVA, F_2,36_ = 11.758, *P* < 0.001; Fig 1A). As the experiment progressed, the differences in shoot biomass of plants inoculated with MAFF and mutants became greater. At 7 and 8wpi MAFF-inoculated plants were significantly larger than hosts inoculated with STM30 which were significantly greater larger than hosts inoculated with STM6 (7wpi: ANOVA, F_2,42_ = 30.212, *P* < 0.001; 8wpi: ANOVA, F_2,34_ = 20.215, *P* < 0.001; Fig 1A).

To quantify fitness effects on the symbiont, we measured characteristics of nodules that reflect host investment into *in planta* rhizobia. We focused on 6 and 8wpi, time points in which variation in symbiotic benefit was detected and natural senescence of nodules had not occurred. At 6wpi we did not detect differences in average nodule biomass infected with different genotypes (ANOVA, F_2,9_ = 4.543, *P* = 0.054; Fig 1B), At 8wpi, nodules housing MAFF had a greater average biomass than nodules of STM30 which were larger than STM6 (ANOVA, F_2,11_ = 17.156, *P* < 0.001; Fig 1B). To examine the possibility that differences in nodule biomass reflected fitness differences of the bacteria, we also dissected and successfully cultured 86 out of 115 nodules to quantify the viable *in planta* rhizobia (S2 Table). MAFF population sizes estimated from cultured nodules at 6 and 8wpi were significantly greater than STM30 and STM6, while no differences were detected between STM30 and STM6 (6wpi: ANOVA, F_2,38_ = 4.673, *P* < 0.05; 8wpi: ANOVA, F_2,46_ = 11.789, *P* < 0.001; Fig 1C). Thus, under controlled conditions, singly inoculated hosts grew smaller with the less effective rhizobia which were simultaneously reciprocated fewer resources than the more effective genotype.

### Host and symbiont growth under ambient conditions

**Fig 2:**
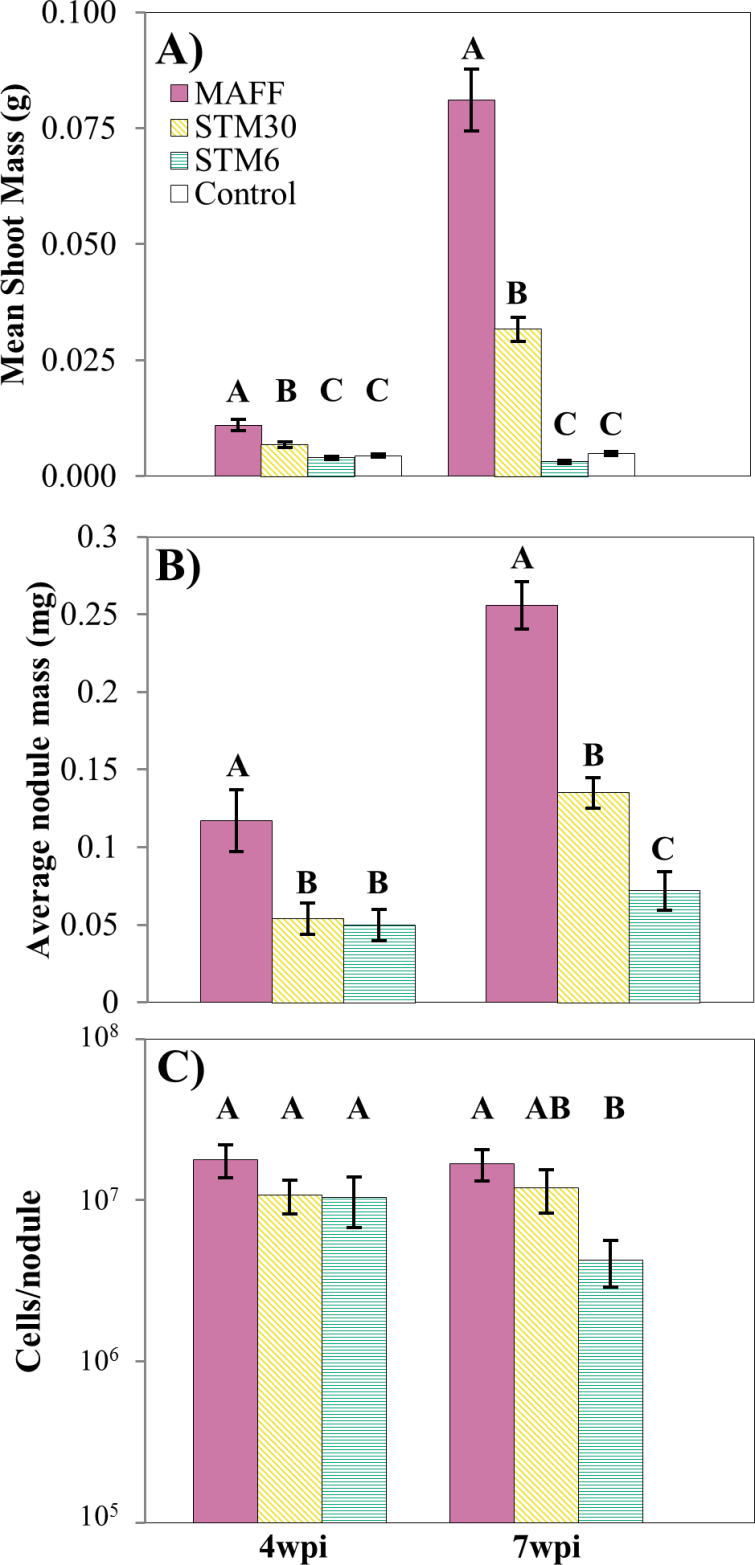
Traits of host and symbiont in greenhouse experiment. *L*. *japonicus* hosts were grown in sterilized quartzite sand supplemented with N-free Jensen’s fertilizer. Hosts were singly inoculated or co-inoculated with the near-isogenic *M*. *loti* strains MAFF, STM30, and STM6 and mean host and symbiont phenotypes were measured. (**A)** Symbiont effectiveness measured as dried shoot biomass. (**B)** Host investment in symbionts was measured as average individual biomass of nodules (dry weight). (**C)** Rhizobial fitness was estimated based on colony forming units (CFUs) from serially diluted extracts of crushed nodules. Error bars indicate one standard error from the mean. Data points demarked with different letters indicate significant differences between strains at a given wpi (one-way ANOVA, post-hoc Student’s t-test, α = 0.05)

Results were repeatable with plants grown in the less controlled environment of greenhouse. In this experiment plants were harvested only at 4wpi and 7wpi, during which plants were rapidly growing and had not flowered. Only two time points were used because of the greater challenges in greenhouse experiments. At 4 and 7wpi hosts inoculated with MAFF had significantly more shoot biomass than those infected with either STM30 or STM6, and plants inoculated with STM30 had significantly greater shoot biomass than those inoculated with the ineffective strain STM6 (4wpi: ANOVA, F_2,35_ = 19.334, *P* < 0.001; 7wpi: ANOVA, F_2,35_ = 89.744, *P* < 0.001; Fig 2A). We also harvested nodules at these times. Again, consistent with results from growth chamber experiments, nodules infected with MAFF had the greatest average biomass at both 4 and 7wpi, while the average biomass of STM30-infected nodules was significantly greater than those from STM6-infected plants at 7wpi (4wpi: ANOVA, F_2,26_ = 6.739, *P* < 0.005; 7wpi: ANOVA, F_2,29_ = 55.839, *P* < 0.001; Fig 2B). Of the 224 nodules cultured, rhizobia were successfully cultured from 177 nodules (S2 Table). Of the nodules harvested at 4wpi, there were no significant differences in the number of cultured rhizobia (ANOVA, F_2,36_ = 0.852, *P* = 0.435; Fig 2C). At 7wpi, MAFF population size was significantly greater than that of STM6, but the population size of STM30 was not different relative to the population sizes of either of the other two genotypes (ANOVA, F_2,40_ = 3.782, *P* < 0.05; Fig 2C).

### Testing models of host control over *in planta* rhizobial fitness

First, we tested whether STM30 or STM6 are compromised for fitness by measuring *in vitro* growth rate and nodulation rate, as these factors could affect the outcome of our experiments. We can reject the possibility that STM30 or STM6 were compromised for our tested fitness measures. The mean doubling times ± standard error in minutes for MAFF, STM30 and STM6 were 210 ± 32, 175 ± 28 and 220 ± 25, respectively, and there were no significant differences in growth rate between the three strains (ANOVA; F_2,32_ = 0.6687; *P* = 0.52). We can also reject the possibility that STM30 or STM6 were compromised for nodulation relative to MAFF nodulation relative to MAFF by comparing early nodulation rates in a single inoculation scenario (4wpi, greenhouse experiment; ANOVA; F_2,35_ = 2.2326; *P* = 0.1232). We co-inoculated 96 greenhouse-grown plants with MAFF paired with each of the less effective genotypes to test models of partner choice and sanctions. The proportion of nodules with MAFF present and the proportion of MAFF within nodules were quantified. We tested the null model that ratios of recovered rhizobia should approximate the ratios used in the initial inoculations, which assumes that nodulation rates and rates of rhizobial *in planta* growth are equal. Under a partner choice model, plants will form more nodules with symbionts that provide greater benefits [24,25]. Under a sanctions model, there will be greater *in planta* proliferation of the more effective genotype[21].

We inoculated plants with a 1:1 and 1:9 (MAFF:STM mutant) ratio of genotypes. A fraction of the mixtures was plated and each genotype was scored based on colony color (the MAFF strain used expressed red fluorescent protein) to retrospectively determine actual proportions used. The proportion of MAFF in MAFF:STM30 mixtures were close to the expected proportions: 0.517 ± 0.030 for the planned 1:1 ratio inoculum and 0.118 ± 0.001 for the planned 1:9 inoculum. In contrast, the proportion of MAFF for the MAFF:STM6 mixtures were more biased towards the mutant than expected and were 0.289 ± 0.032 for the planned 1:1 inoculum and 0.045 ± 0.005 for the 1:9 inoculum. Thus, for the latter combination, the actual ratios of inoculated genotypes were 3:7 and 1:19.

In all but one comparison, MAFF was present in significantly more nodules than the null expectation (Table 1). When MAFF and STM30 were inoculated at equal proportions, MAFF was present in significantly more nodules at 4 and 7wpi (4wpi: two-tailed GoF test, n = 16, *P* < 0.05; 7wpi: two-tailed GoF test, n = 10, *P* < 0.01). The same pattern was observed at 4 and 7wpi when MAFF was co-inoculated at a 3:7 ratio with STM6 (4wpi: two-tailed GoF test, n = 15, *P* < 0.01; 7wpi: two-tailed GoF test, n = 12, *P* < 0.01). Likewise, at both 4 and 7wpi MAFF was present in significantly more nodules than expected when inoculated at a 1:9 ratio with STM30 (4wpi: two-tailed GoF test, n = 11, *P* < 0.01; 7wpi: two-tailed GoF test, n = 9, *P* < 0.01). Additionally, the proportion of nodules with MAFF present was significantly greater than expected at 7wpi (two-tailed GoF test, n = 12, *P* < 0.05), but not 4wpi (two-tailed GoF test, n = 12, *P* = 1), when in a mixed inoculum of 1:19 with STM6. These data are inconsistent with predictions of the null model and suggest that even at the earliest stages of infection *L*. *japonicus* can bias infections rates towards the more beneficial symbiont.

**Table 1:** Host control phenotypes when inoculated with two symbionts.

Harvest	Proportion of MAFF^a^ when coinoculated with near-isogenic mutants (STM30 or STM6)	Proportion of nodules with MAFF present on co-inoculated hosts (partner choice)^b^	Proportional population size of MAFF in nodules of co-inoculated hosts (sanctions)^c^	Fraction of co-infected nodules^d^
**4wpi**	0.5 (STM30)	0.813^e^	0.827±0.169	3/16
	0.3 (STM6)	0.867^f^	0.680±0.231	3/15
	0.1 (STM30)	0.455^f^	0.509±0.176	4/11
	0.05 (STM6)	0.083	0.0001±0.0001^f^	1/12
**7wpi**	0.5 (STM30)	1^f^	0.765±0.214	2/10
	0.3 (STM6)	0.667^f^	0.840±0.121^e^	2/12
	0.1 (STM30)	0.556^f^	0.459±0.154	2/9
	0.05 (STM6)	0.25^e^	0.161±0.093	2/12

^a^ Proportion of MAFF in inocula were used as null expectations

^b^ Observed proportion of nodules with MAFF present on co-inoculated hosts analyzed with a binomial goodness of fit test

^c^ Observed±one standard error from the mean proportion of viable MAFF in nodules of co-inoculated hosts analyzed with a one-sample t-test

^d^ Proportion of co-infected nodules pooled by treatment and harvest

*P-values* are indicated with ^e^(*P* < 0.05) and ^f^(*P* < 0.01)

In all but the 1:19 MAFF:STM6 (4wpi) treatment, MAFF achieved a higher *in planta* population size than the paired mutant (Table 1). But this pattern was only significant in nodules of plants infected with a 3:7 ratio of MAFF:STM6 at 7wpi (one-sample t-test, t = 4.444, df = 3, *P* < 0.05). Additionally, at 4wpi in plants inoculated with a 1:19 ratio of MAFF:STM6, MAFF was estimated at significantly lower than expected proportions (one-sample t-test, t = 499, df = 2, *P* < 0.01). These results are inconsistent with the null model and suggest that the host can efficiently sanction ineffective strains, but the efficacy of sanctions may be lower with mediocre strains or when the beneficial strain is greatly outnumbered.

**Fig 3:**
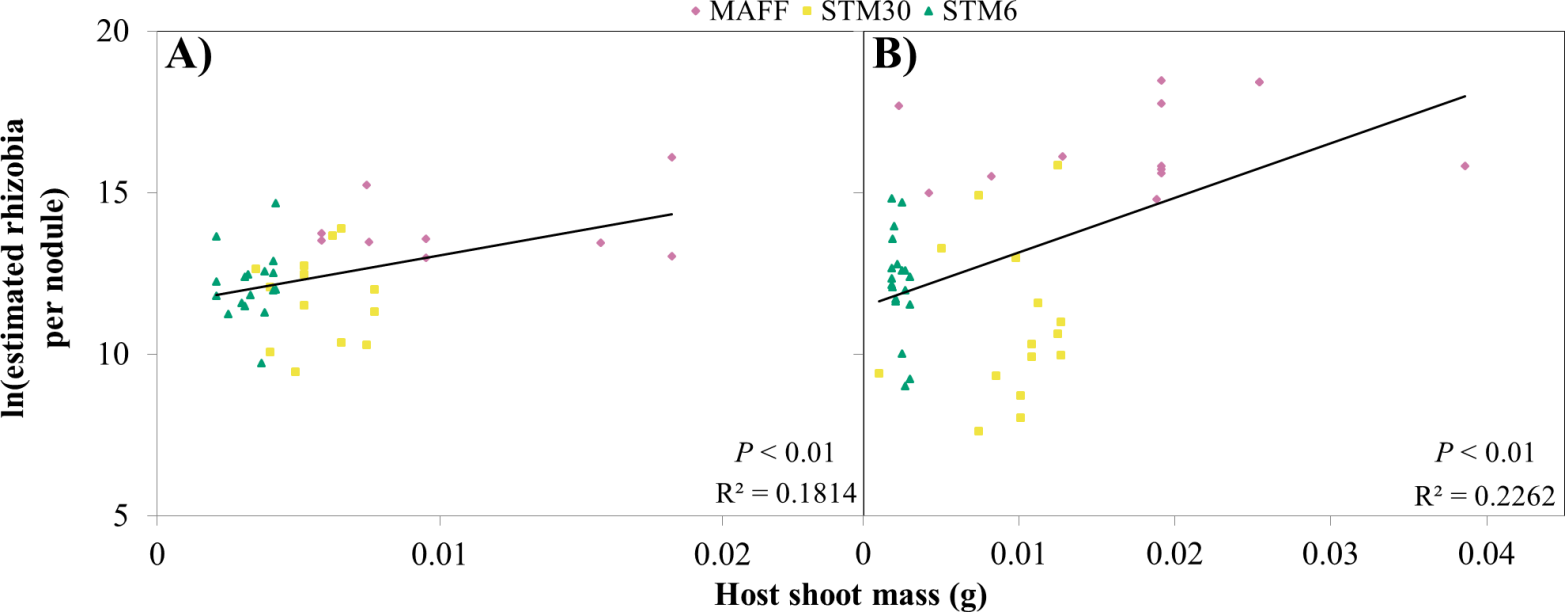
Sanctions are triggered continuously with nitrogen fixation. Linear regression of the natural log of estimated rhizobia per nodule (counting CFUs of serially diluted extracts of crushed nodules) against the shoot biomass of the host(dry weight) Hosts were singly inoculated with near-isogenic *M*. *loti*: MAFF (pink diamond), STM30 (yellow square) and STM6 (green triangle). Each symbol represents one nodule. (**A)** Hosts harvested at 6wpi; F_1,38_ = 8.197. (**B)** Hosts harvested at 8wpi; F_1,46_ = 13.1525.

Models assume that sanctions are either triggered when fixed nitrogen is below a minimal threshold (e.g., sanctioning only of ineffective strains) or are scaled linearly according to the amount of fixed nitrogen [22]. To test these models, we investigated the relationship between symbiotic effectiveness and rhizobial fitness *in planta*. There was a significant correlation between shoot biomass and estimated rhizobial population size, at 6 and 8wpi of plants grown in the growth chamber (6wpi: R^2^ = 0.181, F_1,38_ = 8.197, *P* < 0.01; 8wpi: R^2^ = 0.226, F_1,46_ = 13.1525, *P* < 0.01; Fig 3). While a similar pattern was observed for plants grown in the greenhouse, we could not detect a statistically significant correlation (4wpi: R^2^ = 0.087, F_1,37_ = 3.4437, *P* = 0.07; 7wpi: R^2^ = 0.047, F_1,40_ = 1.9376, *P* = 0.17). These results suggest that the host alters *in planta* fitness of symbionts in a continuous fashion dependent on symbiotic effectiveness.

## Discussion

Using the *L*. *japonicus-M*. *loti* symbiosis we found support for three key hypotheses about host control in legumes: (i) host plants bias nodulation rates against less beneficial rhizobia, (ii) hosts bias *in planta* fitness against less beneficial rhizobia when singly infected and co-infected, and (iii) hosts adjust the severity of sanctions based on symbiotic effectiveness.

Consistent with partner choice theory [25,47], MAFF was significantly enriched, relative to the less-effective STM30 or STM6 near-isogenic mutants, in nodules of plants that had received mixed-strain inocula (Table 1). Given that near-isogenic pairs of symbionts were tested, we suggest that *L*. *japonicus* can detect and respond to differences in symbiotic effectiveness during the early stages of nodule formation. These results are similar to those in which mutant strains varying in nitrogen fixation led to biased infection towards the effective strain [33,35]. However, four other experiments found, regardless of whether hosts formed determinant or independent nodules, no such evidence [33,34,36,37,39,40]. One possible confounding factor is that the mutations introduced in the strains of previous studies had pleiotropic effects on growth and the ability to compete for nodulation sites. STM30 and STM6 were not compromised in *in vitro* growth nor in their ability to nodulate plants, when clonally infected. Furthermore, the use of a mediocre but a near-isogenic mutant (STM30) allowed us to test sanctions hypotheses in a scenario that is more similar to nature, while limiting confounding genetic differences among strains.

Our data suggest that *L*. *japonicus* has a mechanism to detect symbiotic quality before nodule maturation is complete. Histological data of nodule primordia has exhibited phenotypes consistent with a hypersensitive response found in pathogenic infections when symbiont exopolysaccharide genes were mutated [26,27,29], symbionts were maladapted for the inoculated host [41,42,64], or the host was satiated for nitrogen [28,30]. Because nitrogen fixation is not expressed at the time of infection, we hypothesize that the hosts are aborting some nodules before we assayed plants. A mechanism of nodule abortion could explain why the less beneficial genotypes were consistently present in fewer nodules than expected when strains differed in their capacity to fix dinitrogen. However more work is needed to understand the capacity for legumes to detect fixed nitrogen provided by their symbionts, to uncover the cellular and molecular mechanisms for this detection, and to understand how these mechanisms have evolved.

Consistent with models of sanctions [17,21,22], *L*. *japonicus* can alter the *in planta* proliferation of *M*. *loti* dependent on the symbiotic benefit provided. Given that the host has no method to calibrate the benefit received and no alternative choice for receiving fixed nitrogen, it has been debated whether legumes can impose host control when inoculated with a single genotype of symbiont [65–67]. Here, evidence of sanctions was derived from both single inoculation and co-inoculation experiments (Fig 2; Table 1). Moreover, data suggested that sanctions in *L*. *japonicus* are triggered in a continuous fashion and are scaled to the nitrogen fixed by each rhizobial genotype. Similar results were obtained from experiments in which *Glycine max* had received variable rhizobial benefit, which was artificially manipulated by adjusting the amounts of dinitrogen available for fixation [51], and *Acmispon strigosus* (formerly *L*. *strigosus*) inoculated with natural strains that varied in nitrogen fixation [52].

In both natural and agricultural soils, legumes invariably encounter multiple rhizobial genotypes [3–16]. Previous models have suggested that legumes impose sanctions at the level of the whole nodule [17,22], and that this is dependent upon the production of fixed nitrogen regardless of whether it is a clonal or mixed infection. But our data adds to the growing body of literature that suggests hosts can sanction rhizobia at a finer scale within individual nodules. In co-inoculated hosts we consistently found mixed nodules in which MAFF was present at a greater proportion than expected of the null model (Table 1)–(15 out of 19 mixed nodules; S2 Table). These data are consistent with other studies that have found sanctions to be effective within determinate nodules of *A*. *strigosus* [45,46,68].

Evidence for whole nodule sanctions has been found in single inoculation experiments with *Pisum sativa and M*. *truncatula* that inhibited nitrogen fixation with the use of argon in place of dinitrogen [54]. However, it has recently been suggested that indeterminate nodules of *P*. *sativa* [40] and *M*. *sativa* [69] can be infected by multiple genotypes and the less effective strains have decreased *in planta* fitness. Unlike determinate nodules studied here, indeterminate nodules grow indefinitely and have a spatial gradation of rhizobia in different developmental stages [2]. This mixture of rhizobia at various life stages, including nitrogen fixing bacteroids that are terminally differentiated (in most cases; [53]), makes a within nodule mechanism of sanctions more difficult to envision. If an indeteminate nodule is infected by a symbiont that can fix nitrogen, it is only the rhizobia that have terminally differentiated that are providing benefit, while the undifferentiated rhizobia are not. Under this scenario if nitrogen fixation is the trigger for sanctions the host would wrongfully target undifferentiated beneficial symbionts, unless the viable bacteria are able to suppress mechanisms of sanctions, which has been suggested to involve functional nitrogenase [70]. Furthermore, if a nodule is mixed the host would be unable to distinguish symbiont quality of undifferentiated rhizobia, thus targeting rhizobia based on nitrogen fixation would not effectively decrease the relative fitness of less beneficial strains. More research is needed on sanctions in hosts that form indeterminate nodules to examine whether the mechanisms of sanctions are independent from the patterns we have described for species that form determinate nodules.

Historically, scientists have tried to leverage this symbiosis to improve agronomically important leguminous crops, but native low quality symbionts often outcompete elite rhizobial inocula [6,71,72]. Co-inoculation experiments are important because they model the multistrain competition for infections that occur in nature [73], and also because they can enhance our understanding of the effects of nodule co-infection [40,45,46,69,74]. Accordingly, researchers that study sanctions should not neglect the role mixed nodules could play in this complex symbiosis and its evolutionary trajectory. Early models of sanctions focused on a whole nodule mechanism, but if sanctions occur at a finer scale many of these models could be adapted to the plant cell level, or smaller [17,22]. This study demonstrates the utility of combining single inoculation and co-inoculation experimental designs for future studies of host control as we continue to unravel this symbiosis and its stabilizing mechanisms.

## Methods

### Biological materials

*L*. *japonicus* ecotype MG-20 seeds were acquired from LegumeBase (University of Miyazaki, Japan). MG-20 hosts were grown for one generation at the University of California, Riverside (UCR) to generate seeds for this experiment. Seeds were germinated on N-free Jensen’s [63] agar plates (1.5% w/v) prior to planting in one gallon pots filled with a plaster sand and peat moss mixture supplemented with nitrogen, phosphorus, potassium, calcium and trace minerals (UCR soil mix #3). Plants were grown under ambient light from July to October in an insect free greenhouse and watered with a 1:100 dilution of Peters Excel 21-5-20 (Scotts Professional, Marysville, Ohio, USA).

We used a MAFF strain with DsRed integrated into the genome (red fluorescent protein visible under natural light; M. Hayashi, personal communication), andtwo near-isogenic mutants STM30 and STM6, previously generated by signature tagged mutagenesis [61]. We acquired the rhizobial strains from LegumeBase. Bacteria were grown in a liquid medium of Modified Arabinose Gluconate (MAG, 29°C, 180rpm; Sachs et al. 2009).

### *In vitro* MAFF growth assays

Flasks of MAG were inoculated with MAFF, STM30, or STM6 and grown to log-phase growth. Cell density readings were measured optically on a colorimeter and a subset of readings were confirmed via quantitative plating. Doubling time was calculated by estimating cell density at sequential time points [75] and the three fastest doubling times for each flask were averaged. An average doubling time was calculated for eleven independent flasks of each strain.

### Growth chamber experiment

MG-20 seeds were sterilized in bleach (5.25% sodium hypochlorite) for five minutes, followed by seven one-minute rinses in sterile ddH_2_O. Seeds were nick scarified with a sterile razor blade and immediately placed into sterilized CYG germination pouches (Mega International; Newport, MN, USA) filled with 13ml of sterile N-free Jensen’s fertilizer. Bundles of four pouches containing five or ten seeds each were wrapped in aluminum foil and placed in clear plastic boxes (Sterilite 18058606; 13.1”x7.6”x4.5”). The inoculated pouches were maintained in a controlled growth facility (14h:10h day:night cycle; 17–27°C; relative humidity 31%-65%) and plants were fertilized weekly with 10ml of N-free Jensen’s per pouch for the duration of the experiment. When true leaves formed (ca. two weeks) seedlings were inoculated with 50ul of rhizobia, which had been washed, resuspended in 50ul of sterile ddH_2_O at a density of 10^9^ cells ml^-1^, and dripped directly on to the roots. Rhizobial treatments included MAFF, STM30, and STM6. A negative control, using 50ul sterile ddH_2_O was also included.

Plants were harvested at 3, 5, 6, 7, and 8wpi. Excess fertilizer was removed from pouches and photographs of each plant were taken for reference. Nodules were counted, dissected, and photographed. Shoots, roots, and nodules were separated and dried at 60°C ≥3 days prior to weighing biomass. At 6 and 8wpi 15–21 nodules from each treatment group were randomly selected for quantitative culturing. Uninoculated controls were also harvested at this time.

### Greenhouse experiment

Seeds were sterilized and nicked as described above and germinated in sterile ddH_2_O in the dark at 20°C for ca. one week. Germinated seedlings were planted in sterilized Conetainers (SC10; Steuwe and Sons, Tangent, OR, USA) filled with autoclaved quartzite sand. The sand is inert and offers negligible nutrients for plant growth [15]. Seedlings were initially maintained in a controlled growth facility and watered three times a week until true leaves emerged (ca. two weeks). After true leaves formed, seedlings were fertilized weekly with 5ml of N-free Jensen’s. One day after the second fertilization seedlings were moved to the greenhouse and arranged by size which was determined based on the number of leaves. After three days of acclimation, groups of eight size-matched plants were randomly assigned to one of the following inoculation treatments: sterile ddH_2_O, MAFF, STM30, STM6, MAFF:STM30 (1:1), MAFF:STM6 (3:7), MAFF:STM30 (1:9) and, MAFF:STM6 (1:19). Treatments within a block were randomly assigned to a location in the greenhouse. For each treatment, either 5ml of sterile ddH_2_O or 5ml of washed rhizobial cells (10^8^ cells ml^-1^) were inoculated directly into the sand. The realized ratios were empirically measured by serially diluting each inoculum treatment, spread plating (10^−6^ dilution), and CFUs. Plants were harvested at 4wpi and 7wpi. At harvest, shoots, roots, and nodules were dried at 60°C, ≥3 days prior to weighing biomass. There were 24 blocks in total, 12 for each harvest, with four randomly picked for nodule culturing at each harvest. Four randomly selected plants per treatment per harvest were picked to measure proxies of rhizobial fitness. Four nodules from each selected plant were randomly selected for quantitative culturing.

### Nodule culturing

Nodules were individually surface sterilized in bleach for a minimum of 45 seconds (ca. one minute per mm of nodule diameter), rinsed three times in sterile ddH_2_0, and crushed with a sterile pestle in sterile ddH_2_0. The nodule slurry was serially diluted in sterile ddH_2_O, spread plated on MAG (10^−3^, 10^−5^ dilutions; 1.8% agar w/v) and incubated at 29°C for 3 days. Colonies were counted to estimate rhizobial population sizes within a nodule. Nodules from co-inoculated plants were cultured as above and were used to estimate total rhizobial population size within a nodule and the proportion of MAFF by scoring colony color (i.e., red versus white colonies).

### Data analysis

Dry shoot biomass was used as a proxy for symbiotic effectiveness of each rhizobial strain given that the rhizobia provided the sole external source of nitrogen for growing plants in our experiments [45,52]. We used mean individual nodule biomass as a proxy for host investment. Rhizobial population size within a nodule was used as a proxy for rhizobial fitness. Symbiotic effectiveness, host investment in symbionts, symbiont fitness, and number of nodules formed were compared among treatments using analysis of variance (ANOVA) and post-hoc students t-tests (JMP Pro 12.0.1). At 8wpi in the growth chamber experiment two hosts singly inoculated with MAFF were removed from the analysis due to constrained growth within the pouches.

For each mixed strain treatment we quantified the proportion of cultured nodules occupied by MAFF at each harvest and used a binomial goodness of fit test of the realized inocula ratios against the observed proportions of nodules containing MAFF. Estimated fitness of each strain from nodules of mixed strain treatment hosts was measured and proportion of MAFF was calculated per individual host. Hosts from the same treatment were analyzed with a one-sample t-test using realized inocula ratios as the null expected proportions. To test for a correlation between symbiotic effectiveness and symbiont fitness we performed a linear regression (JMP Pro 12.0.1). To normalize estimates of rhizobial population size per nodule the natural log of population size was used.

## Supporting information

S1 Table Data collected during harvests. ^a^nodules weighed for the “Growh Chamber” experiment were pooled by pouch (A, B, C, D, E).
(XLSX)

S2 Table Estimated rhizobial fitness determined by culturing nodules. ^a^Nodule ID for the “Growth Chamber” and “Greenhouse” experiments are represented by (Harvest)(Inoculant)(Pouch).(Nodule #) and (Plant #).(Nodule #), respectively.
(XLSX)
